# Effects of Sodium-Glucose Transporter 2 Inhibitors (SGLT2-I) in Patients With Ischemic Heart Disease (IHD) Treated by Coronary Artery Bypass Grafting *via* MiECC: Inflammatory Burden, and Clinical Outcomes at 5 Years of Follow-Up

**DOI:** 10.3389/fphar.2021.777083

**Published:** 2021-11-15

**Authors:** Celestino Sardu, Massimo Massetti, Nicola Testa, Luigi Di Martino, Gaetano Castellano, Fabrizio Turriziani, Ferdinando Carlo Sasso, Michele Torella, Marisa De Feo, Gaetano Santulli, Giuseppe Paolisso, Raffaele Marfella

**Affiliations:** ^1^ Department of Advanced Medical and Surgical Sciences, University of Campania Luigi Vanvitelli, Naples, Italy; ^2^ Department of Cardiovascular and Arrhythmias, Campobasso, Italy; ^3^ Department of Cardio-thoracic Surgery, Catholic University of Sacred Heart, Rome, Italy; ^4^ Department of Cardiothoracic Surgery, University of Campania Luigi Vanvitelli, Naples, Italy; ^5^ Department of Advanced Biomedical Sciences, International Translational Research and Medical Education Academic Research Unit (ITME), Federico II University, Naples, Italy; ^6^ Department of Medicine, Division of Cardiology, Albert Einstein College of Medicine, Wilf Family Cardiovascular Research Institute, New York, NY, United States; ^7^ Department of Molecular Pharmacology, Fleischer Institute for Diabetes and Metabolism (FIDAM), Montefiore University Hospital, New York, NY, United States; ^8^ Mediterranea Cardiocentro, Naples, Italy

**Keywords:** type 2 diabetes mellitus, coronary heart disease, coronary artery bypass grafting, sodium-glucose transporter 2 inhibitors, minimally invasive extracorporeal circulation, multi-vessel coronary stenosis, over-inflammation

## Abstract

**Introduction:** Minimally invasive extracorporeal circulation (MiECC) reduced inflammatory burden, leading to best clinical outcomes in patients treated with coronary artery bypass grafting (CABG). Despite this, the patients with type 2 diabetes mellitus (T2DM) vs those without T2DM (non-T2DM) have a worse prognosis, caused by over-inflammation and modulated by sodium-glucose transporter 2 receptors. However, we evaluated the inflammatory burden and clinical outcomes in non-T2DM vs T2DM patients under sodium-glucose transporter 2 inhibitors (SGLT2-I users) vs non-SGLT2-I users at 5 years of follow-up post-CABG *via* MiECC.

**Materials and methods:** In a multicenter study, we screened consecutive patients with indications to receive CABG. The study endpoints were the inflammatory burden (circulating serum levels of tumor necrosis factor-alpha (TNF-α), interleukin 1 and 6 (IL-1 and IL-6), C-reactive protein (CRP), and leucocytes count) and the clinical outcomes at follow-up of 5 years in non-T2DM vs SGLT2-I users, in non-T2DM vs non-SGLT2-I users, and SGLT2-I users vs non-SGLT2-I users.

**Results:** At baseline, and at one year and 5 years of follow-up, the non-T2DM vs SGLT2-I users, non-T2DM vs non-SGLT2-I users, and SGLT2-I users vs non-SGLT2-I users had the lowest values of IL-1, IL-6, and TNF-α (*p* < 0.05). At one year of follow-up, SGLT2-I users vs non-T2DM and non-SGLT2-I users vs non-T2DM users had a higher rate of all deaths, cardiac deaths, re-myocardial infarction, repeat revascularization, and stroke, and of the composite endpoint (*p* < 0.05). In a multivariate Cox regression analysis, the composite endpoint was predicted by IL-1 [2.068 (1.367–3.129)], TNF-α [1.989 (1.081–2.998)], and SGLT2-I [0.504 (0.078–0.861)].

**Conclusion:** In T2DM patients, the SGLT2-I significantly reduced the inflammatory burden and ameliorated clinical outcomes at 5 years of follow-up post-CABG *via* MiECC.

## Introduction

Patients with type 2 diabetes mellitus (T2DM) represent about one-third of patients affected by cardiovascular diseases (CVDs) (Einarson et al., 2018). Coronary heart disease (CHD) is the most common manifestation of cardiovascular disease (CVD) and the primary cause of death in T2DM patients (Einarson et al., 2018). Indeed, insulin resistance and hyperglycemia could lead to endothelial dysfunction and vascular complications *via* over-inflammation, causing a worse prognosis in T2DM than in non-T2DM patients (Sardu et al., 2019a). In addition, T2DM vs non-T2DM patients have a higher rate of multivessel coronary stenosis, which causes plaque rupture, acute intracoronary thrombosis, and adverse clinical events (Marfella et al., 2018a; Marfella et al., 2018b). In this context, coronary artery bypass grafting (CABG) is a recommended revascularization strategy to ameliorate clinical outcomes in T2DM patients with multivessel coronary stenosis (Neumann et al., 2018). CABG could be performed *via* different operative techniques and using external circuits as the cardiopulmonary bypass (CPB) circuit (Neumann et al., 2018). On the other hand, this circuit leads to increased blood contact with the foreign surfaces of the CABG circuit and to the requirement of priming fluid, which could trigger an increased systemic inflammatory response (Wan et al., 1997). This over-inflammatory response could lead to a worse prognosis in CABG-treated patients, and particularly in those with T2DM (Wan et al., 1997; Neumann et al., 2018). In this context, the minimally invasive extracorporeal circulation circuit (MiECC) could reduce perioperative inflammation and ameliorate post-CABG’s clinical outcomes (Anastasiadis et al., 2016). The MiECC includes a closed CPB circuit with biologically inert blood contact surfaces and reduced priming volume, added to a cardioplegic system and to a venous bubble trap/venous air removing device (Anastasiadis et al., 2016). Moreover, the MiECC significantly reduces the inflammatory response during CABG as compared to other extracorporeal circulation circuits (Ohata et al., 2007; Gunaydin et al., 2009). Despite this, a higher percentage of T2DM treated *via* MiECC evidenced a worse prognosis after CABG, and diabetes increased by 1.85 folds the risk of mortality after CABG (Winkler et al., 2017). Indeed, the altered glucose homeostasis and insulin resistance could cause over-inflammation, linked to an increased expression of sodium-glucose transporter 2 receptors, and to worse prognosis post-CABG (Sardu et al., 2019b; Sardu et al., 2021). In this context, in T2DM patients treated with CABG, the sodium-glucose transporter 2 inhibitor (SGLT2-I) “empagliflozin” lead to a profound reduction in cardiovascular and all-cause mortality, hospitalization for heart failure, and incident or worsening nephropathy (Verma et al., 2018). However, the SGLT2-I could be used for the secondary prevention of cardiovascular events after CABG in individuals with T2DM (Verma et al., 2018). On the other hand, little is known about the effects on the glucose homeostasis, over-inflammation, and the clinical outcomes exerted by SGLT2-I in T2DM treated with CABG *via* MiECC (Verma et al., 2018; Sardu et al., 2021). Therefore, in the current research, we hypothesized that the SGLT2-I could modulate/reduce the inflammatory burden in CABG-treated patients *via* MiECC, ameliorating clinical outcomes post-CABG. Thus, here we aimed to evaluate T2DM vs non-T2DM patients and T2DM patients divided in SGLT2-I users vs non-SGLT2-I users, the inflammatory burden at baseline, and at 1 and 5 years of follow-up after CABG *via* MiECC. Finally, in these cohorts, we investigated the clinical outcomes in terms of all death, cardiac death, myocardial infarction, stroke, repeat revascularization, and composite endpoint at 1 and 5 years of follow-up after CABG.

## Materials and Methods

We conducted a multicenter study at the University of Campania Luigi Vanvitelli, Naples, Italy; at Catholic University of Sacred Heart, Rome, Italy; and at Gemelli Molise S.p.a, Campobasso, Italy. The study started in January 2010 and ended in December 2015, and the follow-up duration was 5 years. The study was designed to evaluate, in a cohort of patients divided in non-T2DM vs T2DM, the effects of CABG *via* MiECC in terms of inflammatory burden reduction, and clinical outcomes at 5 years of follow-up. Then the patients with T2DM, according to the chronic hypoglycemic drug therapy at the moment of CABG, were further divided in SGLT2-I users vs non-SGLT2-I users. Thus, we screened consecutive patients with a diagnosis of stable CHD and indication to receive coronary angiography and CABG (Neumann et al., 2018). The diagnosis of stable CHD was made according to international recommendations (Neumann et al., 2018). The study population was then enrolled according to the following inclusion and exclusion criteria:

Inclusion criteria: we enrolled the patients that undergo coronary angiography, and those with luminal stenosis of at least 70% in at least two major coronary arteries or in one coronary artery in addition to a 50% or greater stenosis of the left main trunk were classified as multivessels coronary disease (Neumann et al., 2018). The enrolled patients were aged >18 and <75 years with an indication to receive a CABG for multivessel coronary artery stenosis (Neumann et al., 2018).
Exclusion criteria: we excluded from this study the patients with clinical or laboratory evidence of heart failure with New York Heart Association (NYHA) Class III or IV, and acute myocardial infarction, and patients with the previous CABG, previous stroke, valvular heart defects, and patients who required any concomitant cardiac or vascular procedures (as carotid endarterectomy, valve surgery, etc.), (Wan et al., 1997; Neumann et al., 2018). Thus, we excluded the patients with severe uncontrolled hypertension (blood pressure >200/100 mmHg) or secondary causes of hypertension; patients routinely consuming more than three alcoholic drinks per day (Wan et al., 1997; Neumann et al., 2018); kidney failure with estimated glomerular filtration rate (eGFR) < 30 ml/min/1.73 m2.2, (Verma et al., 2018); patients with inflammatory chronic and rheumatic diseases, and oncological diseases.

However, 648 patients indicated to receive a CABG and were enrolled in the study protocol. Finally, we categorized the patients as non-T2DM vs T2DM. The T2DM was diagnosed by the evidence of fasting glucose ≥126 mg/dl (7 mmol/L), post-prandial glucose ≥200 mg/dl (11.1 mmol/L), and glycated hemoglobin A1c (HbA1c) values ≥ 6.5% on two separate tests, according to international recommendations (American Diabetes Associa, 2020). Then the T2DM patients answered a specific questionnaire about the use of SGLT2-I before the beginning of the study, the beginning and the end of treatment, the administration route, and the duration of use (American Diabetes Associa, 2020). Thus, the T2DM patients who never used SGLT2-I were classified as “non-SGLT2-I users.” The T2DM patients who had already used SGLT2-I for at least 6 months were classified as “SGLT2-I users.” The patients SGLT2-I users received either 10 mg or 25 mg of empagliflozin once daily. The cohort of 64 patients SGLT2-I users was recruited at Policlinico Gemelli to receive a CABG treatment for CHD. However, these patients were not randomized to the SGLT2-I therapy in our study. Although SGLT2-I has been present in the European drug market since 2015, the SGLT2-I user patients recruited from 2010 to 2015 were the ones included in authorization randomized trials running in Italy in that period. Notably, these patients (SGLT2-I users) were still treated by SGLT2-I independently by marketing, as usually happens for ethical reasons. In addition, due to the absence of any observed adverse event, those patients did not discontinue the SGLT2-I therapy but underwent CABG for CHD when required. Local Ethical Committee of participating Institutions approved the study (number 29738), and informed written consent was obtained for each patient enrolled. The study was performed in accordance with the Declaration of Helsinki. The study endpoints were evaluated in the study cohorts after CABG at 1 year and 5 years of follow-up. The supplementary files are the full description of the intervention (CABG and MiECC), laboratory analysis, and echocardiographic evaluation.

### Clinical Visits, Data Collection, and Analysis

We evaluated at baseline and follow-up the study population’s clinical characteristics as non-T2DM vs T2DM patients SGLT2-I users, non-T2DM vs T2DM patients non-SGLT2-I users, and T2DM patients SGLT2-I users vs T2DM patients non-SGLT2-I users. The data were collected at baseline and follow-up after clinical discharge by the treating physician, telephonic interviews, hospital admissions, and discharge schedules (Marfella et al., 2018a; Marfella et al., 2018b; Sardu et al., 2019b; Sardu et al., 2021). However, the physicians evaluated each patient’s clinical status, and performed a physical examination to collect vital signs and adverse events (Marfella et al., 2018a; Marfella et al., 2018b; Sardu et al., 2019b; Sardu et al., 2021). Thus, we evaluated the adherence to drug therapy in the study cohorts and any clinical symptom referred by any patient (Marfella et al., 2018a; Marfella et al., 2018b; Sardu et al., 2019b; Sardu et al., 2021). Therefore, we evaluated the clinical outcomes at follow-up end, collecting the data prospectively from electronic medical records used in the clinical setting at participants’ institutions. We used an electronic system to capture, collect, and monitor the data, with on-site and real-time data entry. Finally, the authors collected the patients’ files in each participating Institution that were then analyzed.

### Study Endpoints

The study endpoints were the evaluation of the inflammatory markers and the clinical outcomes at follow-up of 5 years in non-T2DM vs SGLT2-I users, in non-T2DM vs non–SGLT2-I users, and in SGLT2-I users vs non–SGLT2-I users. The inflammatory markers were evaluated by the assay of the circulating serum levels of TNF-α, IL-1, IL-6, CRP, and leucocytes count. The clinical outcomes were any cause death, cardiac death, non-fatal myocardial infarction, stroke, repeat revascularization, and finally, the composite endpoint (the sum of any clinical outcome).

Cardiac death was defined by the evidence of a death event caused by a primary cardiac event/disease (Hicks et al., 2017). However, in cardiac death the primary cause, as the underlying disease or injury that initiated the train of events resulting in death, was represented by an acute myocardial infarction (MI), a fatal arrhythmia, a sudden cardiac death, death due to heart failure, and death due to cardiovascular procedures and hemorrhage, or due to other cardiovascular causes (Hicks et al., 2017).

Non-fatal MI was diagnosed as MI that did not cause a death event (Hicks et al., 2017). Thus, the diagnosis of non-fatal MI was confirmed by the evidence of an acute myocardial injury with clinical evidence of acute myocardial ischemia (Lang et al., 2015) and the increase and/or fall of the cardiac troponin (cTn) values with at least one value above the 99th percentile upper reference limit (URL), (Hicks et al., 2017).

We diagnosed the stroke by the evidence of a central nervous system infarction, defined as brain, spinal cord, or retinal cell death, and attributable to ischemia, and based on neuropathological, neuroimaging, and/or clinical evidence of permanent injury (Hicks et al., 2017). However, the neurological deficit persisted ≥24 h and was attributed to an acute focal injury of the central nervous system by a vascular cause, including cerebral infarction, intracerebral hemorrhage, and subarachnoid hemorrhage (Hicks et al., 2017).

Repeat revascularization was defined by the evidence of a repeat percutaneous coronary intervention, or re-operative bypass graft placement for partial revascularization during the index procedure, or re-operative bypass graft placement for restenosis at the lesion treated during index CABG (Hicks et al., 2017).

### Statistical Analysis

For the statistical analysis, we used the SPSS version 23.0 (IBM statistics). The categorical variables were presented as number and percentage, while continuous variables as either mean ± standard deviation or median and interquartile range, in the case of not normally distributed variables. The normal/not normal distribution was preliminarily assessed through the Kolmogorov–Smirnov Goodness-of-Fit K-S test. One-way analysis of variance (ANOVA) was used to compare baseline data. Then we used the Bonferroni test to evaluate the comparison between the groups of study.

A multivariable logistic regression model was developed from the predicted probabilities of predicting any cause of composite endpoint. The model was adjusted for study variables as age, sex, body mass index (BMI), current smoking, hypertension, dyslipidemia, and smoking history.

The rates of any cause of death, cardiac death, non-fatal myocardial infarction, stroke, and repeat revascularization were derived as Kaplan–Meier estimates and compared by log-rank test at one and 5 years of follow-up. Overall survival and event-free survival were assessed by Kaplan–Meier survival curves and compared by the log-rank test. The resulting hazard ratios (HRs) and 95% CIs were reported. Two-tailed *p*-values < 0.05 were considered statistically significant.

## Results

The study population was represented by 648 consecutive patients with IHD, and treated by a CABG *via* MiECC. According to admission glycemia and DM diagnostic criteria (American Diabetes Associa, 2020), the patients were then divided in 460 non-T2DM vs 188 T2DM patients. However, as previously reported and according to the previous 6 months’ treatment with SGLT2-I the patients were divided in SGLT2-I users (n 64) vs non-SGLT2-I users (n 124). The clinical characteristics of the study population are reported in Table 1.

**TABLE 1:** Characteristics of study population at baseline.

Baseline	Non-T2DM (n 460)	SGLT2-I users (n 64)	Non-SGLT2-I users (n 124)	*p*-value
Age (years)	71.6 ± 10.5	70.6 ± 9.2	70.9 ± 9.7	0.906
Body mass index (Kg/m^2^)	27.1 ± 3.08	28.9 ± 4.7	28.4 ± 4.6	0.030*; 0.031*; 0.989
Male gender, n (%)	254 (55.2)	41 (64.1)	77 (62.1)	0.311
Current smoking, n (%)	239 (52)	30 (46.9)	59 (47.6)	0.413
Diabetes mellitus, n (%)	—	64 (100)	124 (100)	—
Hypertension, n (%)	384 (83.5)	55 (86)	107 (86.3)	0.702
Dyslipidemia, n (%)	344 (74.8)	49 (76.6)	96 (77.4)	0.426
Previous myocardial infarction, n (%)	92 (20)	14 (21.9)	30 (24.2)	0.543
Previous stroke, n (%)	35 (7.6)	6 (9.3)	11 (8.9)	0.756
Peripheral vascular disease, n (%)	28 (6.1)	5 (7.8)	10 (8.1)	0.342
Chronic obstructive pulmonary disease, n (%)	23 (5)	4 (6.2)	7 (5.6)	0.640
Systolic blood pressure, mmHg	130.4 ± 6.0	132.1 ± 3.8	131.6 ± 5.9	0.649
Diastolic blood pressure, mmHg	80.1 ± 3.7	81.2 ± 4.9	81.6 ± 5.4	0.518
Heart rate, bpm	88.1 ± 6.7	88.8 ± 5.8	89.5 ± 5.6	0.427
Glycemia, mg/dL	88.2 ± 4.7	137.5 ± 18.9	139.9 ± 19.8	0.001*; 0.001*; 0.203
Hb1Ac, %	5.2 ± 0.3	6.6 ± 0.9	6.7 ± 0.8	0.001*; 0.001*; 0.362
Cholesterol, mg/dl	206.3 ± 19.2	222.7 ± 20.1	218.2 ± 24.8	0.001*; 0.003*; 0.067
LDL-cholesterol, mg/dl	122.7 ± 20.6	141.2 ± 21.1	138.6 ± 22.3	0.005*; 0.032*; 0.031
Creatinine clearance	70.5 ± 25.7	83.3 ± 21.3	82.8 ± 22.1	0.001*; 0.001*; 0.899
Hb, g/dL	13.9 ± 0.64	11.8 ± 1.12	11.7 ± 1.4	0.526
PLTx10^3^	208.2 ± 59.3	194.5 ± 48.6	196.4 ± 44.5	0.366
Hs-cTnT, mg/dL	154.3 ± 24.3	156.3 ± 23.5	153.2 ± 24.7	0.353
Fibrinogen, mg/dL	463.71 ± 91.65	450.16 ± 119.81	454.41 ± 107.43	0.939
EuroSCORE	3.3 ± 2.3	3.4 ± 2.4	3.4 ± 2.5	0.346
NYHA 1	193 (42)	26 (40.6)	50 (40.3)	0.780
NYHA 2	187 (40.6)	27 (42.2)	51 (41.1)	—
NYHA 3	64 (13.9)	9 (13.9)	17 (13.8)	—
NYHA 4	16 (3.5)	2 (3.1)	6 (4.8)	—
Procedural data	—	—	—	—
Complete revascularization, (%)	299 (65)	41 (64.1)	79 (63.7)	0.933
LIMA use (%)	418 (90.9)	58 (90.6)	113 (91.1)	0.734
Radial use (%)	78 (17)	10 (15.3)	20 (16.1)	0.821
RIMA use (%)	43 (9.3)	6 (9.4)	11 (8.9)	0.951
Number of total conduits	3.0 ± 0.6	3.1 ± 0.5	3.1 ± 0.6	0.518
Number of arterial conduits	1.8 ± 0.9	1.9 ± 0.8	1.9 ± 0.9	0.513
Number of venous conduits	1.3 ± 0.9	1.3 ± 1.2	1.3 ± 1.4	0.522
Left main disease (%)	89 (19.3)	13 (20.3)	24 (19.3)	0.955
Echocardiographic parameters	—	—	—	—
LVTDd, cm	5.43 ± 0.41	5.56 ± 0.39	5.52 ± 0.46	0.576
LVTSd, cm	3.34 ± 0.45	3.35 ± 0.42	3.33 ± 0.48	0.640
LVEF, (%)	54.9 ± 6.3	54.6 ± 6.1	54.8 ± 6.5	0.543
LAD, cm	3.9 ± 0.6	3.8 ± 0.7	4.0 ± 0.5	0.343
Inflammatory markers	—	—	—	—
Lymphocytes, x10^3^	2.721 ± 0.451	2.996 ± 0.561	2.338 ± 0.312	0.568
CRP, mg/dL	0.63 ± 0.28	0.75 ± 0.37	0.76 ± 0.21	0.456
IL-1, mg/dL	4.08 ± 0.83	4.35 ± 0.78	4.76 ± 1.1	0.001*; 0.001*; 0.034**
IL-6, mg/dL	3.23 ± 0.40	4.01 ± 0.27	4.32 ± 0.41	0.016*; 0.002*; 0.049**
TNF-α, mg/dL	5.42 ± 1.05	6.06 ± 0.74	6.83 ± 1.01	0.013*; 0.001*; 0.046**
Medical therapy	—	—	—	—
Aspirin	420 (91.3)	59 (92.2)	114 (91.9)	0.616
Thienopyridine	416 (90.4)	58 (90.6)	112 (90.3)	0.906
Statin	376 (81.7)	53 (82.8)	102 (82.2)	0.594
Beta blocker	331 (71.9)	47 (73.4)	91 (73.4)	0.764
ACEi/ARB, n (%)	359 (78)	51 (79.7)	100 (80.6)	0.608
Loop diuretics, n (%)	99 (21.5)	15 (23.4)	28 (22.6)	
Thiazides, n (%)	59 (12.8)	8 (12.5)	16 (12.9)	0.997
Insulin therapy, n (%)	—	18 (28.1)	36 (29)	0.915
Metformin therapy, n (%)	—	37 (57.8)	69 (55.6)	0.328
Thiazolidinedione, n (%)	—	12 (18.8)	24 (19.3)	0.889
Sulfonylurea, n (%)	—	34 (53.1)	67 (54)	0.851
Incretin, n (%)	—	17 (26.6)	37 (29.8)	0.642
SGLT2-I, n (%)	—	64 (100)	—	—

Non-T2DM, patients without type 2 diabetes mellitus; SGLT2-I, sodium-glucose transporter two inhibitors; Hb1Ac, glycated hemoglobin 1Ac; LDL, low-density lipoprotein; Hb, hemoglobin; PLT, platelets; hs-cTnT, high sensitivity cardiac troponin T; NYHA, New York Heart Association; LIMA, left internal mammary artery; RIMA, right internal mammary artery; LVEDd, left ventricle end-diastolic diameter; LVESd, left ventricle end-systolic diameter; LVEF, left ventricle ejection fraction; LAD, left atrium diameter; CRP, C reactive protein; IL-1, interleukin 1; IL-6, interleukin 6; TNF-α, tumor necrosis factor-alpha; ACEi/ARB, angiotensin-converting enzyme inhibitors/angiotensin receptor blockers.

* is for *p* < 0.05 vs non-T2DM.

** is for *p* < 0.05 comparing SGLT2-I users vs non-SGLT2-I users.

**At baseline**, SGLT2-I users vs non-T2DM, and non-SGLT2-I users vs non-T2DM had higher values of BMI, glycemia, Hb1Ac, total cholesterol, and LDL-cholesterol (*p* < 0.05), and lower values of creatinine clearance (*p* < 0.05) Table 1. Non-T2DM vs SGLT2-I users, and non-T2DM vs non-SGLT2-I users had the lowest values of IL-1, IL-6, and TNF-α (*p* < 0.05); these inflammatory markers were over-expressed in non-SGLT2-I users as compared to SGLT2-I users (*p* < 0.05) Table 1. At baseline, no other significant differences were found between cohorts of study. Table 1.

### Inflammatory Markers Expression

**FIGURE 1:**
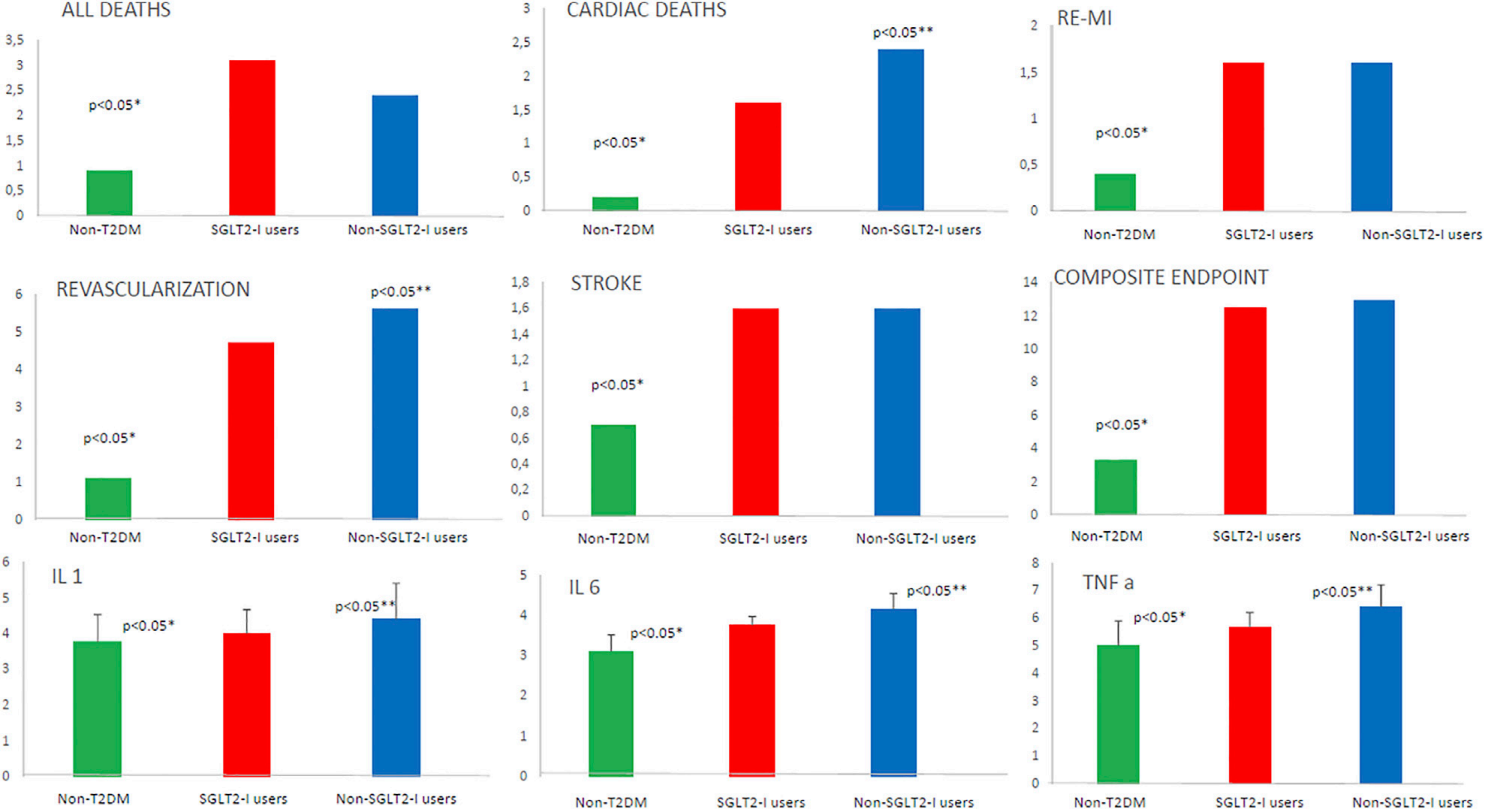
The representation of the study outcomes at 1 year of follow-up in patients without type 2 diabetes mellitus (non-T2DM; green color) vs T2DM under sodium-glucose transporter 2 inhibitors (SLGT2-I users; red color) vs T2DM patients without sodium-glucose transporter 2 inhibitors (non-SLGT2-I users; blue color). We reported the percentage of events for all deaths, cardiac deaths, re-myocardial infarction (re-MI), stroke, revascularization, and composite endpoint. For interleukin 1 (IL-1), interleukin 6 (IL-6), and tumor necrosis factor-alpha (TNF-α), we used the values as mean ± standard deviation. * Is for statistical significant (*p*-value <0.05) vs T2DM; ** is for statistical significant (*p* value < 0.05) comparing SGLT2-I users vs non-SLGT2-I users.

**FIGURE 2:**
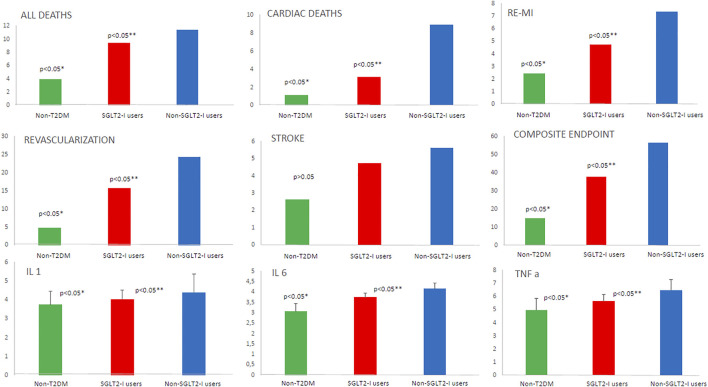
The representation of the study outcomes at 5 years of follow-up in patients without type 2 diabetes mellitus (non-T2DM; green color) vs T2DM under sodium-glucose transporter 2 inhibitors (SLGT2-I users; red color) vs T2DM patients without sodium-glucose transporter 2 inhibitors (non-SLGT2-I users; blue color). We reported the percentage of events for all deaths, cardiac deaths, re-myocardial infarction (re-MI), stroke, revascularization, and composite endpoint. For interleukin 1 (IL-1), interleukin 6 (IL-6), and tumor necrosis factor-alpha (TNF-α), we used the values as mean ± standard deviation. * Is for statistical significant (*p*-value < 0.05) vs T2DM; ** is for statistical significant (*p* value < 0.05) comparing SGLT2-I users vs non–SLGT2-I users.

At one year of follow-up after CABG, the non-SGLT2-I users over-expressed, compared to SGLT2-I users and non-T2DM, the serum inflammatory markers (IL-1, IL-6, and TNF-α; *p* < 0.05). Table 2 and Figure 1. These serum inflammatory markers were higher in SGLT2-I users as compared to non-T2DM patients (*p* < 0.05). Table 2 and Figure 1. The same trend regards the over-inflammatory burden was confirmed at 5 years of follow-up in the study cohorts Table 2 and Figure 2.

**TABLE 2:** Study outcomes: inflammatory markers and clinical outcomes at 1, and 5 years of follow-up.

One year of follow-up					5 years of follow-up		—	
—	Non-T2DM (n 460)	SGLT2-I users (n 64)	Non-SGLT2-I users (124)	*p*-value	Non-T2DM (n 460)	SGLT2-I users (n 64)	Non-SGLT2-I users (124)	*p*-value
Inflammatory markers								
Lymphocytes, x10^3^	2.460 ± 0.422	2.523 ± 0.326	2.538 ± 0.312	0.150; 0.108; 0.353	2.387 ± 0.415	2.518 ± 0.303	2.535 ± 0.308	0.141; 0.890; 0.326
CRP, mg/dL	0.58 ± 0.22	0.65 ± 0.30	0.66 ± 0.24	0.118; 0.083; 0.915	0.58 ± 0.22	0.64 ± 0.22	0.65 ± 0.39	0.202; 0.126; 0.915
IL-1, mg/dL	3.77 ± 0.76	4.01 ± 0.66	4.41 ± 1.0	0.001*; 0.001*; 0.005*	3.73 ± 0.72	4.02 ± 0.49	4.38 ± 0.99	0.001*; 0.001*; 0.005*
IL-6, mg/dL	3.11 ± 0.40	3.75 ± 0.22	4.16 ± 0.38	0.006*; 0.001*; 0.005*	3.06 ± 0.38	3.74 ± 0.20	4.15 ± 0.28	0.001*; 0.001*; 0.022*
TNF-α, mg/dL	5.01 ± 0.89	5.68 ± 0.53	6.43 ± 0.78	0.004*; 0.001*; 0.006*	4.94 ± 0.89	5.63 ± 0.50	6.45 ± 0.82	0.001*; 0.001*; 0.001*
CLINICAL OUTCOMES	—	—	—	—	—	—	—	—
All deaths, n (%)	4 (0.9)	2 (3.1)	3 (2.4)	0.001*; 0.005*; 0.194	18 (3.9)	6 (9.4)	14 (11.3)	0.004*; 0.006*; 0.001**
Cardiac deaths, n (%)	1 (0.2)	1 (1.6)	3 (2.4)	0.015*; 0.015*; 0.048*	5 (1.1)	2 (3.1)	10 (8.9)	0.001*; 0.001*; 0.001*
Re-myocardial infarction, n (%)	2 (0.4)	1 (1.6)	2 (1.6)	0.034*; 0.034*; 0.852	11 (2.4)	3 (4.7)	9 (7.3)	0.030*; 0.047*; 0.008*
Stroke, n (%)	3 (0.7)	1 (1.6)	2 (1.6)	0.041*; 0.041*; 0.852	12 (2.6)	3 (4.7)	7 (5.6)	0.152; 0.181; 0.055
Revascularization, n (%)	5 (1.1)	3 (4.7)	7 (5.6)	0.005*; 0.001*; 0.045*	21 (4.6)	10 (15.6)	30 (24.2)	0.001*; 0.001*; 0.001*
Composite endpoint, n (%)	15 (3.3)	8 (12.5)	16 (12.9)	0.001*; 0.001*; 0.674	67 (14.6)	24 (37.5)	70 (56.4)	0.001*; 0.001*; 0.001*

Non-T2DM: non-diabetics; SGLT2-I: sodium-glucose trasporter two inhibitors; CRP: C reactive protein; IL-1: interleukin 1; IL-6: interleukin 6; TNFα: tumor necrosis alpha.

* is for statistical significant (*p* value < 0.05) vs non-T2DM.

** is for statistical significant (*p* value < 0.05) comparing SGLT2-I users vs non-SGLT2-I users.

### Study Clinical Outcomes

At 1 year of follow-up, we evidenced in SGLT2-I users vs non-T2DM, and in non-SGLT2-I users vs non-T2DM a higher rate of all deaths, cardiac deaths, re-myocardial infarction, repeat revascularization, stroke, and composite endpoint (*p* < 0.05). Table 2 and Figure 1. Intriguingly, non-SLGT2-I users vs SGLT2-I users evidenced a higher rate of cardiac deaths and repeat revascularization (*p* < 0.05). Table 2 and Figure 1.

At 5 years of follow-up, non-SGLT2-I users, compared to SGLT2-I users and to non-T2DM patients, had a higher rate of all clinical study endpoints (*p* < 0.05), except that for stroke Table 2 and Figure 2. Notably, the same trend was observed comparing SGLT2-I users vs non-T2DM patients (*p* < 0.05). Table 2 and Figure 2.

In a multivariate Cox regression analysis, the composite endpoint was predicted by IL-1 [2.068, CI 95% (1.367–3.129)], TNF-α values [1.989, CI 95% (1.081–2.998)], and by SGLT2-I therapy [0.504, CI 95% (0.078–0.861)]. Table 3.

**TABLE 3:** Multivariate Cox regression analysis for clinical outcomes at 5 years of follow-up.

Risk factors	Multivariate analysis for the composite endpoint		—
	HR	CI 95%	*p*-value
Age	0.718	0.028-1.130	0.210
Gender	3.331	0.662-9.519	0.152
BMI	1.010	0.855-1.193	0.906
Heart rate	0.771	0.637-1.933	0.224
Smoking	1.417	0.586-3.427	0.439
Diabetes	1.044	0.160-6.828	0.964
Dyslipidemia	1.477	0.431-5.062	0.535
Hypertension	1.413	0.193-10.357	0.733
Previous MI	0.480	0.164-4.401	0.179
NYHA class III	0.845	0.337-2.188	0.720
LVEF	0.993	0.921-1.071	0.851
Clearance creatinine	1.006	0.974-1.040	0.698
EuroSCORE	2.504	0.980-3.102	0.122
Leucocytes	1.198	0.703-2.042	0.505
IL-1	2.068	1.367-3.129	0.001*
TNF-α	1.989	1.081-2.998	0.012*
Fibrinogen	0.993	0.986-1.001	0.079
Insulin therapy	0.963	0.390-6.301	0.305
Beta-blockers	1.153	0.533-2.498	0.119
SGLT2-I	0.504	0.078-0.861	0.047*

HR, Hazard ratio; CI, confidence of interval; BMI, Body mass index; MI, myocardial infarction; NYHA, New York Heart Association; LVEF, left ventricle ejection fraction; IL-1, interleukin 1; TNF-α, tumor necrosis alpha; SGLT2-I, sodium-glucose transporter two inhibitor.

* is for statistical significant (*p* < 0.05).

**FIGURE 3:**
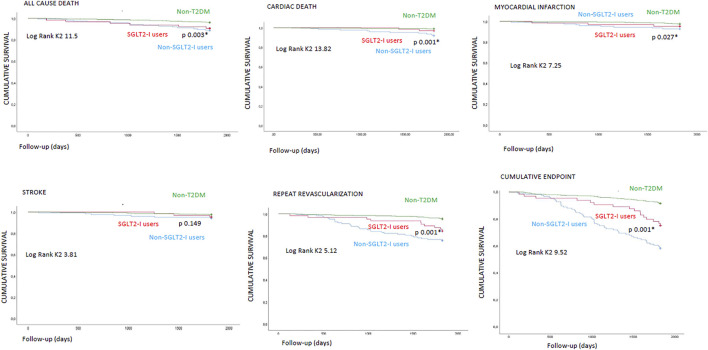
Kaplan survival curves in non-diabetics (non-T2DM) vs type 2 diabetes mellitus (T2DM) patients under sodium-glucose transporter 2 inhibitors (SGLT2-I users) vs T2DM patients without SGLT2-I (non-SGLT2-I users) for study clinical outcomes. * is for statistical significant (*p* < 0.05).

The Kaplan curves showed the cumulative survival free from study endpoints in the study cohorts at 5 years of follow-up. Figure 3.

## Discussion

In the present study, we evaluated the effects of MiECC in terms of significantly reducing the inflammatory burden and ameliorating the clinical outcomes in non-T2DM vs T2DM patients treated by CABG. Notably, and clinically relevant, T2DM vs non-T2DM over-expressed at baseline, and at 5 years of follow-up, the inflammatory cytokines (IL-1, IL-6, TNF-α). The over-inflammation was more significant in T2DM cohorts of non-SGLT2-I users as compared to SGLT2-I users’ patients. Finally, the non-SGLT2-I users vs SGLT2-I users (vs non-T2DM patients) experienced a worse prognosis, as a higher rate of deaths, cardiac deaths, HF events, strokes, repeat revascularization, and of the composite endpoint.

The MiECC has been validated as a technique superior to standard extracorporeal circulation in lower mortality and myocardial damage, with improved end-organ protection and easy application (Puehler et al., 2011; Kowalewski et al., 2016). Notably, the lower rate of mortality observed in patients receiving a MiECC was mainly due to the modulation/reduction of inflammatory burden and complement activation response. Indeed, the MiECC reduced inert surfaces of the closed extracorporeal circulation system and the priming volume. These effects minimized hemodilution and could influence the onset of anticoagulation disorders (van Boven et al., 2013). On the other hand, the patients with altered glucose homeostasis and insulin resistance evidenced over-inflammation, increased expression of sodium-glucose transporter 2 receptors, and worse prognosis after CABG (Sardu et al., 2021). In detail, the patients with higher levels of inflammatory cytokines evidenced the over-expression of the sodium-glucose transporter 2 receptors al level of peri-coronary fat (Sardu et al., 2021). Intriguingly, the metformin therapy in these patients ameliorated the glucose homeostasis and insulin resistance, with consequent downregulation of the inflammatory cytokines and of the sodium-glucose transporter 2 receptors al level of the peri-coronary fat excised during CABG (Sardu et al., 2021). Furthermore, the significant peri-coronary downregulation of the sodium-glucose transporter 2 receptors, linked to the best clinical outcomes post-CABG (Sardu et al., 2021). In our study, we evidenced, at baseline, the over-inflammation (higher serum values of IL-1, IL-6, and TNF-α) in T2DM vs non-T2DM patients, and much more in non-SGLT2-I users vs SGLT2-I users. In this context, the MiECC could significantly reduce inflammatory cytokines (serum values of IL-1, IL-6, and TNF-α) in non-T2DM and in SGLT2-I users vs non-SGLT2-I users’ patients treated with CABG. Notably, the over-inflammation is a well-known negative factor influencing clinical outcomes and worse prognosis in CABG patients receiving a MiECC (Puehler et al., 2011; van Boven et al., 2013). Indeed, in our study, the highest values of IL-1 and of TNF-α increased the risk to have the composite endpoint of study (worse prognosis) of 2.068 and 1.989 times, respectively. Thus, we confirm the over-inflammation as the most significant and negative prognostic factor in CHD patients with T2DM (Gallinoro et al., 2021; Paolisso et al., 2021). On the contrary, we investigated that the SGLT2-I could reduce the risk of having the study’s composite endpoint (worse prognosis) about the 50%. This ameliorative effect could be the result of anti-inflammatory properties of SGLT2-I, added to pleiotropic clinical effects *via* the down expression of sodium-glucose transporter 2 receptors in CABG patients (Verma et al., 2018; Sardu et al., 2021).

Moreover, here we first reported the systemic anti-inflammatory effects of SGLT2-I in T2DM patients treated with CABG *via* MiECC. Second, we found that anti-inflammatory effects *via* the block of sodium-glucose transporter 2 receptors/pathways induced by the SGLT2-I therapy could lead to the best clinical outcomes and the increased ameliorative effects of the MiECC in the T2DM patients (Puehler et al., 2011; van Boven et al., 2013; Kowalewski et al., 2016). Indeed, over-inflammation and T2DM are two main and independent risk factors leading to a worse prognosis in CABG-treated patients receiving a MiECC. However, the SGLT2-I therapy could enhance the anti-inflammatory effects of MiECC in CABG patients with T2DM, and leading to the best clinical outcomes. Recently, authors investigated that the inhibition of SGLT2 by empagliflozin reduced the inflammatory/oxidative stress in the non-infarcted myocardium of rats (Oshima et al., 2018). Thus, it reduced the mortality post-MI, acting by the protective modification of cardiac energy metabolism and antioxidant proteins in the diabetic heart (Oshima et al., 2018). Similarly, the SGLT2-I canagliflozin caused either a glucose-independent upregulation of cardiac survival pathways leading to cardioprotective effects in high-risk cardiovascular patients irrespective of diabetic status (Lim et al., 2019). Finally, among the T2DM patients, the current vs non-SGLT2-I users presented a significantly lower rate of major adverse cardiac events in 2 years following endarterectomy (D'Onofrio et al., 2021). However, this could confirm the critical involvement of the SGLT2 in the inflammatory process of diabetic atherosclerotic lesions and suggest its possible favorable modulation by SGLT2-I that could lead to the best clinical outcomes (D'Onofrio et al., 2021).

Therefore, we might speculate that SGLT2-I could have anti-inflammatory effects linked to the downregulation of the SGLT2 expression in humans treated with CABG *via* MiECC. However, SGLT2-I could exert a protective role in humans with T2DM and treated *via* MiECC, by the significant downregulation of the inflammatory axis, and leading to best clinical outcomes.

### Study Limitations

The current investigation evidenced few study limitations. First, the cohort of SGLT2-I users is represented by 64 patients with T2DM. Thus, the small dimension of the sample size could affect the clinical outcomes. Second, all study cohorts received the MiECC. The MiECC was used in a previous study conducted in the overall population and in T2DM patients treated by CABG (Puehler et al., 2011; van Boven et al., 2013; Kowalewski et al., 2016; Winkler et al., 2017). Therefore, we did not match a group of patients treated with conventional extracorporeal circulation in the present study. To date, this could be limiting for a definitive conclusion about the MiECC effect in CABG patients. Finally, the T2DM patients were under chronic SGLT2-I therapy, so they were not randomized to the SGLT2-I therapy. This could limit the current study results. Notably, it could furnish us with a real picture of patients with T2DM under a chronic SGLT2-I therapy. To date, it could limit the bias from the randomization and masking of drug therapy. However, the best evidence would be guaranteed by performing a prospective randomized controlled multicenter study. Therefore, further studies in a larger population, at a more extended time follow-up duration, are needed to investigate all these molecular, cellular, and clinical effects in DM patients under SGLT2-I, and referred for CABG *via* MiECC.

## Conclusion

Taken together, our data could indicate that chronic SGLT2-I therapy could exert ameliorative effects in T2DM receiving a CABG *via* MiECC. This effect is played by systemic anti-inflammatory properties of SGLT2-I, *via* the downregulation of SLGT2 receptors. Conversely, in the clinical setting, chronic SGLT2-I therapy resulted in best clinical outcomes at 5 years of follow-up after CABG intervention *via* MiECC.

## Data Availability

The raw data supporting the conclusions of this article will be made available by the authors, without undue reservation.
