# Supplementary material for: Effects of Sodium-Glucose Transporter 2 Inhibitors (SGLT2-I) in Patients With Ischemic Heart Disease (IHD) Treated by Coronary Artery Bypass Grafting via MiECC: Inflammatory Burden, and Clinical Outcomes at 5 Years of Follow-Up
Source: Front Pharmacol. 2021 Nov 15;12:777083. doi: 10.3389/fphar.2021.777083 (PMC8634684; doi:10.3389/fphar.2021.777083)
Supplement: Supplementary file 1 [file DataSheet1.docx]

**Effects of sodium-glucose transporter 2 inhibitors (SGLT2-I) in patients with ischemic heart disease (IHD) and treated by coronary artery bypass grafting via MiECC: inflammatory burden, and clinical outcomes at 5 years of follow-up**

Celestino Sardu, MD, MSc, PhD1,2*; Massimo Massetti, MD, 2,3; Nicola Testa, MD2; Luigi Di Martino, MD2; Gaetano Castellano, MD2; Fabrizio Turriziani, MD1; Ferdinando Carlo Sasso, MD, PhD1; Michele Torella, MD4; Marisa De Feo, MD4; Gaetano Santulli, MD, PhD5,6,7; Giuseppe Paolisso, MD1,8; and Raffaele Marfella, MD, PhD1,8.

1. Department of Advanced Medical and Surgical Sciences, University of Campania “Luigi Vanvitelli”, Naples, Italy; 2. Cardiovascular and Arrhythmias Department, “Gemelli Molise S.p.a”, Campobasso, Italy; 3. Department of Cardio-thoracic surgery”Catholic University of Sacred Heart”, Rome, Italy; 4. Department of Cardio-thoracic Surgery, University of Campania “Luigi Vanvitelli”, Naples, Italy; 5. Department of Advanced Biomedical Sciences, International Translational Research and Medical Education Academic Research Unit (ITME), “Federico II” University, 80131 Naples, Italy; 6. Department of Medicine, Division of Cardiology, Albert Einstein College of Medicine, Wilf Family Cardiovascular Research Institute, New York, NY 10461, USA; 7. Department of Molecular Pharmacology, Fleischer Institute for Diabetes and Metabolism (FIDAM), Montefiore University Hospital, New York, NY 10461, USA; 8. “Mediterranea Cardiocentro”, Naples, Italy.

**Keywords:** type 2 diabetes mellitus, coronary heart disease, coronary artery bypass grafting, sodium-glucose transporter 2 inhibitors, Minimally invasive extracorporeal circulation.

***Corresponding author:**

Celestino Sardu, MD, MSc, PhD address: Piazza Miraglia, 2; 80138, Naples, Italy. Telephone: +39 0815665110; fax: +39 0815095303. email: [drsarducele@gmail.com](mailto:drsarducele@gmail.com)

**SUPPLEMENTARY METHODS**

**Intervention: coronary artery bypass grafting and minimally invasive extracorporeal circulation** All the enrolled patients received the coronary artery bypass grafting (CABG) via minimally invasive extracorporeal circulation (MiECC). The CABG was performed as previously described (**1**-**7**), and according to international guidelines for myocardial revascularization (**8**). We used the MiECC closed circuit (Livanova®, Modena, Italy), as an extracorporeal perfusion circuit. This circuit included the oxygenator membrane (A. L. One AF Plus of Eurosets®, Modena, Italy) and the centrifugal pump (RotaFlow, Jostra AG). This circuit did not have an open venous reservoir, and all the components of the MiECC were coated with heparin (**2**-**5**). However, we used a priming volume of 600–400 ml to reduce negative side effects (**2**-**5**). To date, we used a single shot (100 ml) crystalloid cardioplegia, with the perfusion flow during MiECC at 2 litres per square meter of body surface area, and optimized by the perfusionist (**2**-**5**). Finally, we administered the unfractionated heparin (200–300 units/kilogram body weight) per institutional protocol as an initial bolus to all patients and tailored to the target of activated clotting time of at least 480 seconds (ACT plus®, Medtronic©).

**Laboratory Analysis**

After an overnight fast, we measured the plasma glucose, glycated hemoglobin A1c (HbA1c), and serum lipid levels by enzymatic assays in the hospital chemistry laboratory. In addition, we collected venous blood samples for troponin I (Behring Diagnostics, Westwood, Massachusetts) in ethylene diamine tetra acetic acid-coated tubes before CABG. Troponin T was measured with an Opus Magnum device (Behring Diagnostics), with a discriminator value recommended by the manufacturer. Then, we determined the levels of fasting blood glucose before CABG. Fasting and postprandial plasma glucose data were obtained from the average of each assessment. From peripheral venous samples, we evaluated the inflammatory pro-inflammatory cytokines as the tumor necrosis factor-α, (TNF α), the interleukin-1 (IL1), the interleukin-6, (IL6), the systemic inflammatory markers as the C reactive protein, (CRP), and the leucocytes and neutrophils count (**6**, **7**). The laboratory analysis exams were performed at baseline and at 1 and 5 years of follow-up in the study cohorts.

**Echocardiographic evaluation**

Two experienced physicians in echocardiography and blinded to the study protocol performed a trans-thoracic two-dimensional echocardiogram with M-mode recordings, conventional Doppler, and pulsed-wave tissue Doppler imaging (TDI) measurements. The exams were performed at baseline (before CABG) and at 1 and 5 years of follow-up in the enrolled patient using a Philips iE33 echocardiography (Eindhoven, The Netherlands). We acquired the echocardiographic images in the parasternal long and short-axis views, with the calculation of the LV end-diastolic diameter (LVEDD), end-systolic diameter (LVESD), and the LVEF via the Simpson method (**9**). The echocardiographic measurements were systematically averaged in five consecutive samples.

**References**

1. Wan S, LeClerc JL, Vincent JL. Inflammatory response to cardiopulmonary bypass: mechanisms involved and possible therapeutic strategies. Chest. 1997 Sep;112(3):676-92. DOI: 10.1378/chest.112.3.676.
2. Anastasiadis K, Murkin J, Antonitsis P, Bauer A, Ranucci M, Gygax E, Schaarschmidt J, Fromes Y, Philipp A, Eberle B, Punjabi P, Argiriadou H, Kadner A, Jenni H, Albrecht G, van Boven W, Liebold A, De Somer F, Hausmann H, Deliopoulos A, El-Essawi A, Mazzei V, Biancardi F, Fernandez A, Weerwind P, Poehler T, Derrick C, Waanders F, Gunaydin S, Ohri S, Gummert J, Angelini G, Falk V, Carrel T. Use of minimal invasive extracorporeal circulation in cardiac surgery: principles, definitions and potential benefits. A position paper from the Minimal invasive Extracorporeal Technologies international Society (MiECTiS). Interact Cardiovasc Thorac Surg. 2016 May;22(5):647-62. DOI: 10.1093/icvts/ivv380.
3. Ohata T, Matsuno M, Yamamura M, et al. Minimal cardiopulmonary bypass attenuates neutrophil activation and cytokine release in coronary artery bypass grafting. J Artif Organs 2007;10:92-5. 10.1007/s10047-007-0377-0.
4. Gunaydin S, Sari T, McCusker K, et al. Clinical evaluation of minimized extracorporeal circulation in high-risk coronary revascularization: impact on air handling, inflammation, hemodilution and myocardial function. Perfusion 2009;24:153-62.
5. Winkler B, Heinisch PP, Zuk G, Zuk K, Gahl B, Jenni HJ, Kadner A, Huber C, Carrel T. Minimally invasive extracorporeal circulation: excellent outcome and life expectancy after coronary artery bypass grafting surgery. Swiss Med Wkly. 2017 Jul 10;147:w14474. DOI: 10.4414/smw.2017.14474.
6. Sardu C, D'Onofrio N, Torella M, Portoghese M, Loreni F, Mureddu S, Signoriello G, Scisciola L, Barbieri M, Rizzo MR, Galdiero M, De Feo M, Balestrieri ML, Paolisso G, Marfella R. Pericoronary fat inflammation and Major Adverse Cardiac Events (MACE) in prediabetic patients with acute myocardial infarction: effects of metformin. Cardiovasc Diabetol. 2019 Sep 30;18(1):126. doi: 10.1186/s12933-019-0931-0.
7. Sardu, C.; D’Onofrio, N.; Torella, M.; Portoghese, M.; Mureddu, S.; Loreni, F.; Ferraraccio, F.; Panarese, I.; Trotta, M.C.; Gatta, G.; Galdiero, M.; Sasso, F.C.; D’Amico, M.; De Feo, M.; Balestrieri, M.L.; Paolisso, G.; Marfella, R. Metformin Therapy Effects on the Expression of Sodium-Glucose Cotransporter 2, Leptin, and SIRT6 Levels in Pericoronary Fat Excised from Pre-Diabetic Patients with Acute Myocardial Infarction. Biomedicines 2021, 9, 904. https://doi.org/10.3390/biomedicines9080904.
8. Neumann FJ, Sousa-Uva M, Ahlsson A, Alfonso F, Banning AP, Benedetto U, Byrne RA, Collet JP, Falk V, Head SJ, Jüni P, Kastrati A, Koller A, Kristensen SD, Niebauer J, Richter DJ, Seferovic PM, Sibbing D, Stefanini GG, Windecker S, Yadav R, Zembala MO; ESC Scientific Document Group. 2018 ESC/EACTS Guidelines on myocardial revascularization. Eur Heart J. 2019 Jan 7;40(2):87-165. DOI: 10.1093/eurheartj/ehy394. Erratum in: Eur Heart J. 2019 Oct 1;40(37):3096.
9. Roberto M. Lang, Luigi P. Badano, Victor Mor-Avi, Jonathan Afilalo, Anderson Armstrong, Laura Ernande, Frank A. Flachskampf, Elyse Foster, Steven A. Goldstein, Tatiana Kuznetsova, Patrizio Lancellotti, Denisa Muraru, Michael H. Picard, Ernst R. Rietzschel, Lawrence Rudski, Kirk T. Spencer, Wendy Tsang, Jens-Uwe Voigt. Recommendations for Cardiac Chamber Quantification by Echocardiography in Adults: An Update from the American Society of Echocardiography and the European Association of Cardiovascular Imaging. Journal of the American Society of Echocardiography, Volume 28, Issue 1, 2015,Pages 1-39.e14; DOI: 10.1016/j.echo.2014.10.003.
